# Increased HERV-E clone 4–1 expression contributes to DNA hypomethylation and IL-17 release from CD4^+^ T cells via miR-302d/MBD2 in systemic lupus erythematosus

**DOI:** 10.1186/s12964-019-0416-5

**Published:** 2019-08-14

**Authors:** Xin Wang, Chaoshuai Zhao, Chengzhong Zhang, Xingyu Mei, Jun Song, Yue Sun, Zhouwei Wu, Weimin Shi

**Affiliations:** Department of Dermatology, Shanghai General Hospital, Shanghai Jiaotong University School of Medicine, 100 Haining Road, Shanghai, 200080 China

**Keywords:** *HERV-E clone 4–1*, Systemic lupus erythematosus, Transcription factors, DNA hypomethylation, *miR-302d*, *MBD2*

## Abstract

**Background:**

Increased human endogenous retroviruses E clone 4–1 (HERV-E clone 4–1) mRNA expression is observed in systemic lupus erythematosus (SLE) patients and associates with the disease activity. In this study, we want to further investigate the mechanism of HERV-E clone 4–1 mRNA upregulation and its roles in SLE progression.

**Methods:**

CD4^+^ T cells were isolated from venous blood of SLE patients or healthy controls and qRT-PCR was used to detect HERV-E clone 4–1 mRNA expression. We then investigated the regulation of Nuclear factor of activated T cells 1 (NFAT1) and Estrogen receptor-α (ER-α) on HERV-E clone 4–1 transcription and the functions of HERV-E clone 4–1 3′ long terminal repeat (LTR) on DNA hypomethylation and IL-17 release.

**Results:**

We found HERV-E clone 4–1 mRNA expression was upregulated in CD4^+^ T cells from SLE patients and positively correlated with SLE disease activity. This is associated with the activation of Ca^2+^/calcineurin (CaN)/NFAT1 and E2/ER-α signaling pathway and DNA hypomethylation of HERV-E clone 4–1 5’LTR. HERV-E clone 4–1 also takes part in disease pathogenesis of SLE through miR-302d/Methyl-CpG binding domain protein 2 (MBD2)/DNA hypomethylation and IL-17 signaling via its 3’LTR.

**Conclusions:**

HERV-E clone 4–1 mRNA upregulation is due to the abnormal inflammation/immune/methylation status of SLE and it could act as a potential biomarker for diagnosis of SLE. HERV-E clone 4–1 also takes part in disease pathogenesis of SLE via its 3’LTR and the signaling pathways it involved in may be potential therapeutic targets of SLE.

**Electronic supplementary material:**

The online version of this article (10.1186/s12964-019-0416-5) contains supplementary material, which is available to authorized users.

## Background

Systemic lupus erythematosus (SLE) is an autoimmune disease in which autoreactive CD4^+^ T cells play an important role [1]. Genetic interactions with environmental factors, particularly ultraviolet light exposure, infection and hormonal factors, might initiate the disease, resulting in immune dysregulation at the level of cytokines, T cells, B cells and macrophages [2].

Human endogenous retroviruses (HERV) are descendants of occasional germline invasion by exogenous retroviruses which occupy as much as 8% of the human genome [3]. *HERV-E clone 4–1* is inserted in the short arm of chromosome 19 at position 19p12 upstream of the *ZNF66* gene locus and in the antisense orientation. This full-length HERV-E clone 4–1 is considered to be an LTR2C prototype containing 5′ and 3′ LTR elements that are 95.5% identical and encompass gag, pol and env genes (GenBank: M10976, Additional file 1: Figure S1) [4]. Enhanced expression of mRNA from *HERV-E clone 4–1* was reported in SLE than healthy controls (HCs) [5, 6], and our former study demonstrated that *HERV-E clone 4–1* mRNA expression was increased in SLE patients, and the expression level of *HERV-E clone 4–1* was associated with SLE disease activity index (SLEDAI) [7]. *HERV-E clone 4–1* 5’LTR/LTR2C was hypomethylated in CD4^+^ T cells from SLE patients [7–9] which might have close relationship with its expression.

In this study, we sought to further investigate the mechanism of *HERV-E clone 4–1* mRNA upregulation and its roles in SLE progression, and to estimate the potential value of *HERV-E clone 4–1* in acting as a biomarker and therapeutic target for SLE.

## Methods

### Ethics and selection of patients

This research was approved by the Institutional Research Ethics Committee of Shanghai General Hospital and abided by the ethical guidelines of the Declaration of Helsinki. All the patients involved in this study were adult and written informed consents were obtained from all the patients. All patients with SLE were diagnosed in accordance with the 1997 ACR revised criteria for classification of SLE. Disease activity was assessed using the SLE disease activity index (SLEDAI), and active disease was defined as an SLEDAI score ≥ 5. Age- and sex-matched healthy controls were recruited from the medical staff at Shanghai General Hospital.

### Isolation, culture and treatment of CD4^+^ T cells

Peripheral blood mononuclear cells (PBMC) were isolated from venous blood of SLE patients or healthy controls using Ficoll-paque density gradient centrifugation. Purified CD4^+^ T cells were negatively isolated from PBMCs by CD4^+^ T-cell isolation kits (STEMCELL Technologies, Vancouver, Canada) according to the manufacturer’s protocol. CD4^+^ T cell purity was routinely > 90% as verified through flow cytometry. The cells were then cultured in Xvivo 15 medium (Lonza, Walkersville, MD, USA) supplemented with 10% human AB serum (Valley Biomedical, Winchester, VA, USA) at 37 °C with 5% CO_2_. The treatments of the cells were: TNF-α (HY-P7058, MedChemExpress, NJ, USA), 10 ng/ml, 24 h; IL-6 (HY-P7044, MedChemExpress), 10 ng/ml, 24 h; 17β-estradiol (estradiol/E2) (HY-B0141, MedChemExpress), 100 nmol/L, 24 h; Lipopolysaccharides (LPS) (L8880, Solarbio, Beijing, China), 100 ng/ml, 24 h; ultraviolet B (UVB), 50 mJ/cm2 [10]; hydroxychloroquine sulfate (HCQ sulfate) (HY-B1370, MedChemExpress), 6 μg/ml, 24 h; 5-Azacytidine (5-aza C) (HY-10586, MedChemExpress), 1 mM, 24 h; prednisolone (HY-17463, MedChemExpress), 10 ng/ml, 24 h; AZD9496 (HY-12870, MedChemExpress), 5 nM, 24 h.

### Quantitative reverse transcription-PCR (qRT-PCR)

Total RNAs of cells were extracted using Trizol (Invitrogen) according to the instructions provided by the manufacturer. Reverse transcription was performed using the Primescript RT Master Mix (Takara, Otsu, Japan), and cDNA was amplified using SYBR-Green Premix (Takara). The expression of *HERV-E clone 4–1 gag* was normalized to the expressions of *GAPDH*. The data were analyzed by delta Ct method. Primers of *HERV-E clone 4–1* gag used in this study were imported from other published articles [5–7] and the primers were, F: 5′-CACATGGTGGAGAGTCGTGTTT-3′ and R: 5′-GCTTGCGGCTTTTCAGTATAGG-3′; *GAPDH*, F: 5′-GGAGTCCACTGGCGTCTTC-3′ and R: 5′-GCTGATGATCTTGAGGCTGTTG-3′. Primers for *HERV-E clone 4–1* 3’LTR were, F: 5′-TCGCCACTTCTCCTGTTGTC-3′ and R: 5′-TATTCGGCCGGGATCATTGG-3′.

### Oligonucleotide, plasmids and transfection

SiRNA, *miR-302d* mimics and corresponding negative controls were transfected by Hiperfect transfection reagent (Qiagen, Valencia, CA, USA) and plasmids were transfected by Lipofectamine 3000 (Invitrogen, Carlsbad, CA, USA) into cells. *Nuclear factor of activated T cells 1* (*NFAT1*) siRNA and *Estrogen receptor-α* (*ER-α*) siRNA were obtained from Santa Cruz Biotechnology (sc-36,055, sc-29,305, Santa Cruz, CA, USA). The 3’LTR of *HERV-E clone 4–1* were cloned into pcDNA 3.1 plasmid and the recombinant plasmid was transfected into cells to obtain the 3’LTR mRNA overexpression.

### Western blot analysis

Cells were lysed using radioimmunoprecipitation (RIPA) lysis buffer (Beyotime, Shanghai, China). Protein concentrations were detected using bicinchoninic acid (BCA) Protein Assay Kit (Thermo Fisher Scientific, Rockford, IL, USA). Total proteins were separated by sodium dodecyl sulfate polyacrylamide gel electrophoresis (SDS-PAGE) and transferred onto a polyvinylidene difluoride (PVDF) membrane (Millipore, USA). Antibodies used in the assays were *NFAT1* antibody (ab2722, Abcam, Cambridge, UK), *ER-α* antibody (#8644, Cell Signaling Technology) and GAPDH antibody (#5174, Cell Signaling Technology), *IRF9* antibody (#76684, Cell Signaling Technology), *Methyl-CpG binding domain protein 2* (*MBD2*) antibody (ab38646, Abcam) and IL-17 antibody (ab77171, Abcam).

### Luciferase assay

An NFAT luciferase reporter plasmid (pNFAT-Luc) containing *NFAT1* binding promoter elements was used to detect the *NFAT1* transcriptional activity. CD4^+^ T cells were co-transfected with a mixture of 300 ng pNFAT-Luc reporter and 5 ng pRL-TK Renilla luciferase reporter. After different treatment, the luciferase activities were measured using the Dual Luciferase Reporter assay (Promega, Madison, WI, USA). pRL-TK Renilla luciferase reporter was used to normalize the transfection efficiency.

Full-length sequences of *HERV-E clone 4–1* 5′ LTR containing wild-type of *NFAT1* or *ER-α* predicted binding site was inserted into PGL3-Basic luciferase reporter vector (Promega). Mutant reporter plasmids were prepared using Mutagenesis Kit (Stratagene, La Jolla, CA, USA). Cells were co-transfected with a mixture of 300 ng firefly luciferase reporter, 5 ng pRL-TK Renilla luciferase reporter, and *NFAT1* or *ER-α* plasmids. After 48 h of incubation, the luciferase activities were quantified using the Dual Luciferase Assay System (Promega). The sequences of 3’LTR of *HERV-E clone 4–1* mRNA or *MBD2* 3’UTR containing potential wild-type or mutant binding sites of *miR-302d* were constructed into pmirGLO vectors (Promega). The luciferase vectors and *miR-302d* mimics were transfected into CD4^+^ cells along with pRL-TK vector. The dual-luciferase Reporter assay system (Promega) was used to detect luciferase activity. pRL-TK Renilla luciferase reporter was used to normalize the transfection efficiency.

### Chromatin immunoprecipitation (ChIP)

ChIP assay was conducted using EZ ChIP kit (Millipore, Billerica, MA, USA) and *NFAT1* antibody (ab2722, Abcam) or *ER-α* antibody (#8644, Cell Signaling Technology) according to the instruction of the manufacturer. The primers specific to *HERV-E clone 4–1* 5′ LTR were: 5′-CTCCCCAACCTCCCCTTTTC-3′ and 5′-TGAGAAACATGACTGGGGGC-3′. Normal rabbit IgG (A7016, Beyotime, Shanghai, China) was used to control the nonspecific immunoprecipitation.

### DNA extraction and global methylation analysis

Assays of DNA extraction and global methylation analysis was described in our previous study [11].

### Enzyme-linked immunosorbent assay

The concentration of IL-17 in culture supernatants were measured by Human IL-17 ELISA Kit (ab119535, Abcam) according to the manufacturer’s instructions. Optical density values were read at 450 nm using ELx800 Absorbance Microplate Reader (BioTek, VT, USA).

### Statistical analysis

Statistical analysis was performed using the SPSS program (version 18.0; SPSS, Chicago, IL, USA). The statistical significance of differences between two groups was tested using Student’s t test. Spearman’s analysis was used to test correlation. *P* < 0.05 was considered as statistically significant.

## Results

### *HERV-E clone 4–1* mRNA expression was upregulated in CD4^+^ T cells from SLE patients

**Fig. 1:**
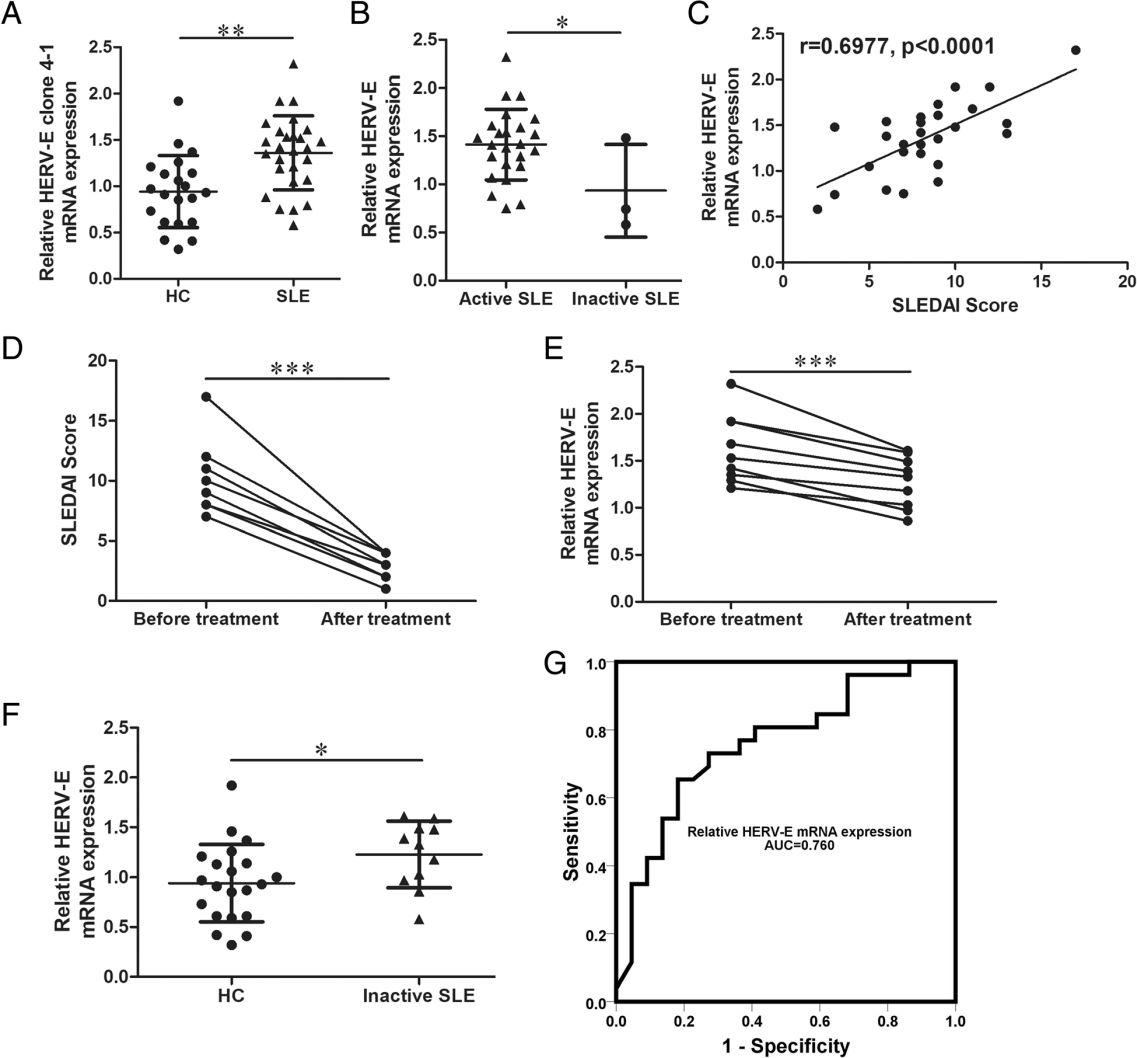
*HERV-E clone 4–1* mRNA expression was upregulated in CD4^+^ T cells from SLE patients. **a** and **b**) Relative expression of *HERV-E clone 4–1* mRNA in CD4^+^ cells from SLE patients (*N* = 27) and healthy controls (HCs) (*N* = 21) or active SLE patients (*N* = 24) and inactive SLE patients (*N* = 3) compared using the unpaired Student’s t test. **c** Correlation between expression of *HERV-E clone 4–1* mRNA and SLEDAI score analyzed with Spearman’s analysis. **d** and **e** SLEDAI score and *HERV-E clone 4–1* mRNA expression in patients who got oral prednisolone and hydroxychloroquine treatment (*N* = 9); data were compared using the paired Student’s t test. **f** Relative expression of *HERV-E clone 4–1* mRNA in CD4^+^ cells from inactive SLE patients (*N* = 11) and HCs (*N* = 21) compared using the unpaired Student’s t test. **g** ROC curve of relative *HERV-E clone 4–1* mRNA expression for differentiating SLE patients from HCs. Data were represented as mean ± SD. *P < 0.05, **P < 0.01, ***P < 0.001

In our former study, we found that *HERV-E clone 4–1* mRNA expression was higher in lupus CD4^+^ T cells than in cells from healthy controls and the *HERV-E clone 4–1* mRNA expression level was positively correlated with SLE disease activity [7]. To continue our study, first, we used new samples (Additional file 1: Table S1) to prove *HERV-E clone 4–1* mRNA expression was higher in SLE CD4^+^ T cells than in cells from healthy controls using Quantitative reverse transcription-PCR (qRT-PCR) (Fig. 1a). We also found that *HERV-E clone 4–1* mRNA expression level was higher in active patients than that of inactive patients (Fig. 1b) and positively correlated with SLE disease activity (Fig. 1c). We also followed-up some patients who got oral prednisolone and hydroxychloroquine treatment and the activity of SLE changed from active to inactive. We found that the *HERV-E clone 4–1* mRNA expressions decreased as the SLEDAI decreased (Fig. 1d and e). But the *HERV-E clone 4–1* mRNA expressions of the inactive patients were also higher than that of HCs (Fig. 1f). What’s more, to assess the diagnostic value of *HERV-E clone 4–1* mRNA for SLE, we performed Receiver Operating Characteristic (ROC) curve analysis to differentiate SLE from HC with the relative *HERV-E clone 4–1* mRNA expressions of the SLE patients and healthy controls (Fig. 1f). The Area Under Curve (AUC) was 0.760, the 95% confidence interval (95% CI) was 0.622 to 0.897, and the best Youden’s index is 0.5. This indicated that *HERV-E clone 4–1* mRNA might have good diagnostic value for SLE and could act as a potential diagnostic biomarker for SLE.

### *NFAT1* activity was increased in SLE and associated with increased *HERV-E clone 4–1* mRNA

**Fig. 2:**
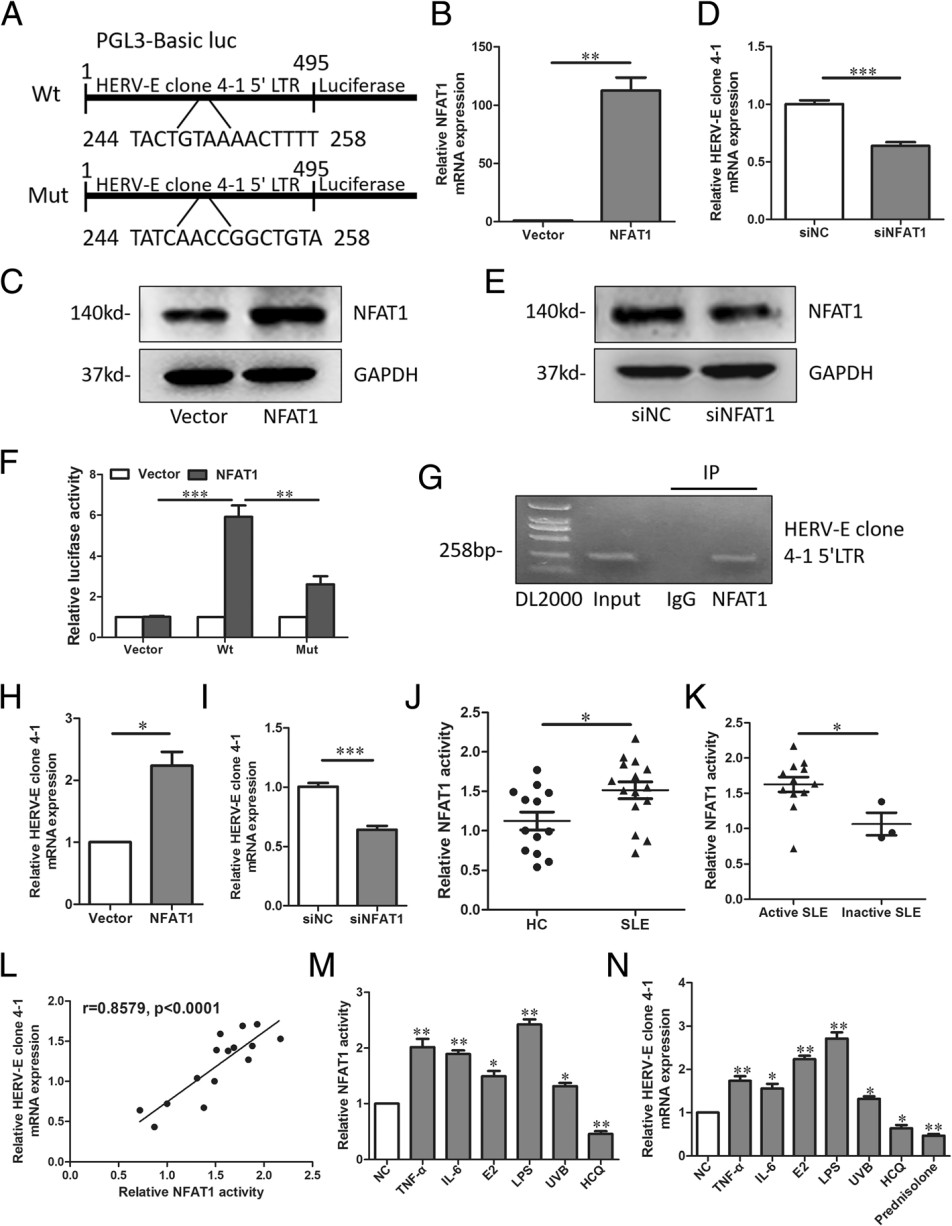
*NFAT1* activity was upregulated in CD4^+^ T cells from SLE patients and closely associated with increased *HERV-E clone 4–1* mRNA expression. **a** Predicted wild-type (wt) binding sites and corresponding mutant (mut) sites of *NFAT1* on *HERV-E clone 4–1* 5’LTR. **b**-**e** qRT-PCR and western-blot assays showing relative *NFAT1* mRNA and protein expression in CD4^+^ cells from SLE patient with *NFAT1* overexpression or knockdown. **f** Luciferase assays were performed in CD4^+^ T cells from SLE patient transfected with wt or mut luciferase reporter. Each luciferase activity was normalized to the value obtained in the cells transfected with vector (N = 3). **g** ChIP assay was used to assess *NFAT1* binding site at *HERV-E clone 4–1* 5’LTR. **h** and **i**) Relative *HERV-E clone 4–1* mRNA expression in CD4^+^ cells from SLE patient with *NFAT1* overexpression or knockdown (*N* = 3). **j** and **k** Relative *NFAT1* activity in CD4^+^ cells from SLE patients (*N* = 15) and HCs (*N* = 13) or active SLE patients (*N* = 12) and inactive SLE patients (*N* = 3) compared using the unpaired Student’s t test. **l** Correlation between relative *NFAT1* activity and relative *HERV-E clone 4–1* mRNA expression analyzed with Spearman’s analysis (*N* = 15). **m** and **n** CD4^+^ T cells from SLE patient were treated with TNF-α, IL-6, E2, LPS, UVB, HCQ or prednisolone in vitro. Relative *NFAT1* activity (L) and relative *HERV-E clone 4–1* mRNA expression (m) were detected. Data were represented as mean ± SD. *P < 0.05, **P < 0.01, ***P < 0.001

To explain why *HERV-E clone 4–1* mRNA was upregulated in CD4^+^ T cells from SLE patients, we wondered if some transcription factors could promote the transcription of *HERV-E clone 4–1* mRNA. Since the 5′ LTR contained the transcription factor binding sites [12], this region was used to predict the potential transcription factors. Using TransFac and JASPAR database, we found some transcription factors that might regulate the expression of *HERV-E clone 4–1* mRNA. *NFAT1*, which was proved to play critical roles in SLE [13] caught our attention. First, full length fragment of the human Human endogenous retroviral DNA (4–1) 5′ LTR with wild type (wt) or mutant (mut) predicted *NFAT1* binding site was inserted into the luciferase reporter plasmid (Fig. 2a). Then, we use *NFAT1* overexpression plasmids to overexpress *NFAT1* and *NFAT1* siRNA to knockdown *NFAT1* (Fig. 2b-e). Luciferase reporter analysis showed that overexpression of *NFAT1* led to an increase in luciferase activity of the wt *HERV-E clone 4–1* 5’LTR plasmid in CD4^+^ T cells, while mut *NFAT1* binding site attenuated the increase of luciferase activity (Fig. 2f). In addition, Chromatin immunoprecipitation (ChIP) assay clearly showed that the predicted *NFAT1*-binding site in *HERV-E clone 4–1* 5′ LTR presented the ability to bind to *NFAT1* protein (Fig. 2g). Moreover, qRT-PCR analysis showed that overexpression of *NFAT1* could increase the expression of *HERV-E clone 4–1* mRNA and knockdown of *NFAT1* with siRNA could decrease the expression of *HERV-E clone 4–1* mRNA (Fig. 2h and i). Then, we collected CD4+ T cells of SLE patients and HCs to detected *NFAT1* activity using NFAT luciferase reporter assay and *HERV-E clone 4–1* mRNA expression. We found *NFAT1* activity was upregulated in CD4+ T cells from SLE patients (Fig. 2j) and higher in active patients than that of inactive patients (Fig. 1k). What’s more, the relative *NFAT1* activity had strong correlation with *HERV-E clone 4–1* mRNA expression (Fig. 2l). So, these results all together suggested that *NFAT1* could induce *HERV-E clone 4–1* mRNA expression via binding to its 5′ LTR. We also detected the influence of some factors on *NFAT1* activity and *HERV-E clone 4–1* mRNA expression (Fig. 2m and n).

### E2 could upregulate *HERV-E clone 4–1* mRNA expression via *ER-α* in CD4^+^ T cells from SLE patients

**Fig. 3:**
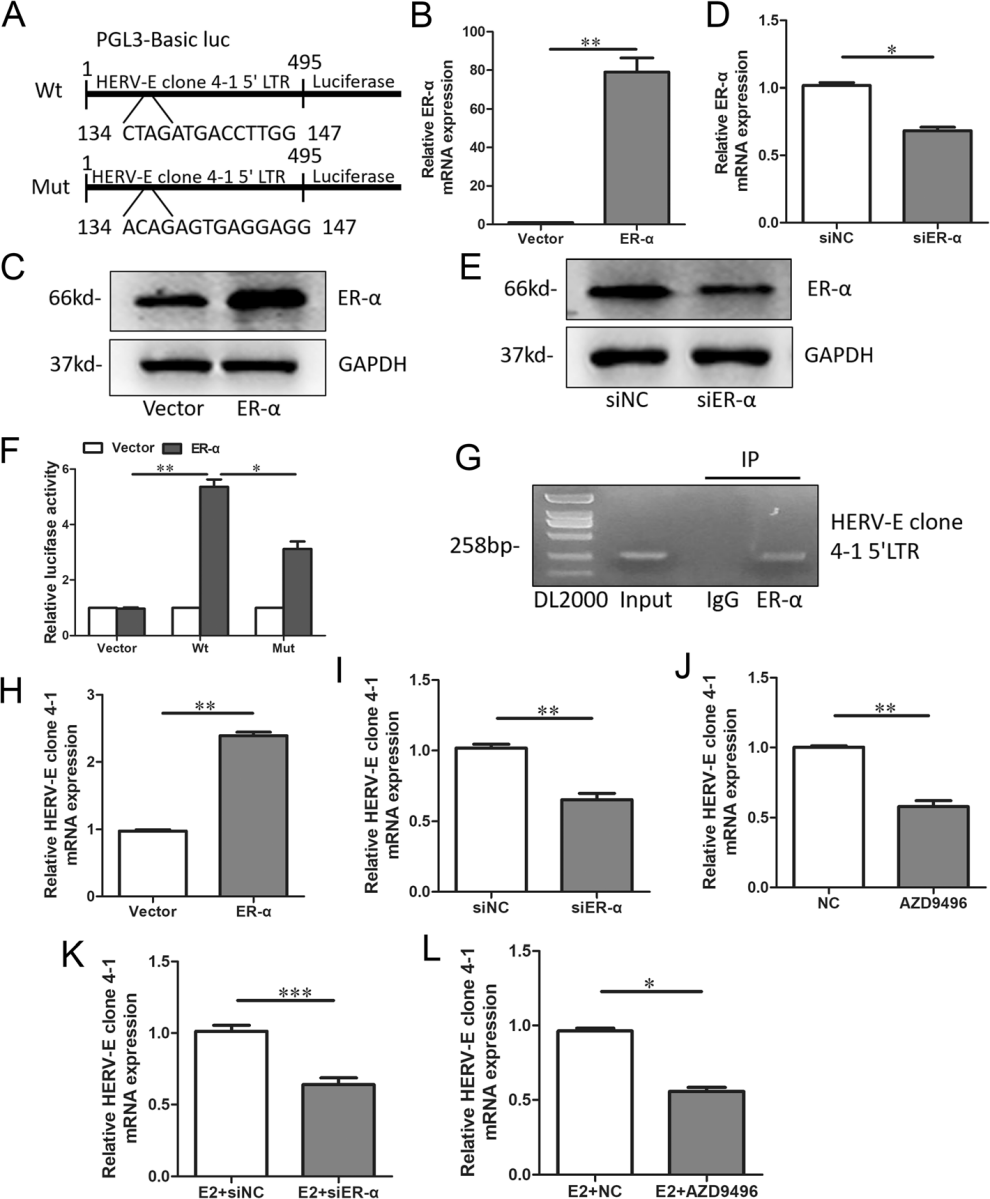
E2 could upregulate *HERV-E clone 4–1* mRNA expression via *ER-α* in CD4^+^ T cells from SLE patients. **a** Predicted wild-type (wt) binding sites and corresponding mutant (mut) sites of *ER-α* on *HERV-E clone 4–1* 5’LTR. **b**-**e** qRT-PCR and western-blot assays showing relative *ER-α* mRNA and protein expression in CD4+ cells from SLE patient with *ER-α* overexpression or knockdown. **f** Luciferase assays were performed in CD4^+^ T cells from SLE patient transfected with wt or mut luciferase reporter. Each luciferase activity was normalized to the value obtained in the cells transfected with vector. **g** ChIP assay was used to assess *ER-α* binding site at *HERV-E clone 4–1* 5’LTR. **h** Relative *HERV-E clone 4–1* mRNA expression in CD4+ cells from SLE patient with *ER-α* overexpression compared using the paired Student’s t test. **i** and **j** Relative *HERV-E clone 4–1* mRNA expression in CD4^+^ cells from SLE patient with *ER-α* inhibition using siRNA or AZD9496 compared using the paired Student’s t test. **k** and **l** Relative *HERV-E clone 4–1* mRNA expression in CD4^+^ cells from SLE patient when *ER-α* siRNA or AZD9496 was used after E2 treatment compared using the paired Student’s t test. Data were represented as mean ± SD, *N* = 3. *P < 0.05, **P < 0.01, ***P < 0.001

When selecting the potential transcript factors that might regulate the expression of *HERV-E clone 4–1* mRNA, *ER-α*, which was the receptor of E2 drew our attention. This is because SLE has a predilection for females of child-bearing age who have relatively high estrogen level and estrogen is also a risk factor for SLE [14] and HERV-E was upregulated in breast cancer and ovarian cancer [15, 16]. Then, we further explored the role of E2 and *ER-α* in SLE. Accordingly, full length fragment of the human endogenous retroviral DNA (4–1) 5′ LTR with wild type (wt) or mutant (mut) predicted *ER-α* binding site was inserted into the luciferase reporter plasmid (Fig. 3a). Then, we use *ER-α* overexpression plasmids to overexpress *ER-α* and *ER-α* siRNA to knockdown *ER-α* (Fig. 3b-e). Luciferase reporter analysis showed that overexpression of *ER-α* led to an increase in luciferase activity of the wt *HERV-E clone 4–1* 5’LTR plasmid in CD4^+^ T cells, while mut *ER-α* binding site attenuated the increase of luciferase activity (Fig. 3f). In addition, ChIP assay clearly showed that the predicted *ER-α*-binding site in *HERV-E clone 4–1* 5′ LTR presented the ability to bind to *ER-α* protein (Fig. 3g). Moreover, qRT-PCR analysis showed that *ER-α* plasmids could increase the expression of *HERV-E clone 4–1* mRNA while *ER-α* antagonist AZD9496 maleate and *ER-α* siRNA could decrease the expression of *HERV-E clone 4–1* mRNA (Fig. 3h-j). In addition, AZD9496 and *ER-α* siRNA could reverse the upregulated *HERV-E clone 4–1* mRNA expression induced by E2 (Fig. 3k and l). So, these results all together suggested that E2 could also upregulate *HERV-E clone 4–1* mRNA expression via *ER-α* in CD4^+^ T cells from SLE patients.

### DNA hypomethylation of *HERV-E clone 4–1* 5’LTR contributed to the increase of *HERV-E clone 4–1* mRNA

**Fig. 4:**
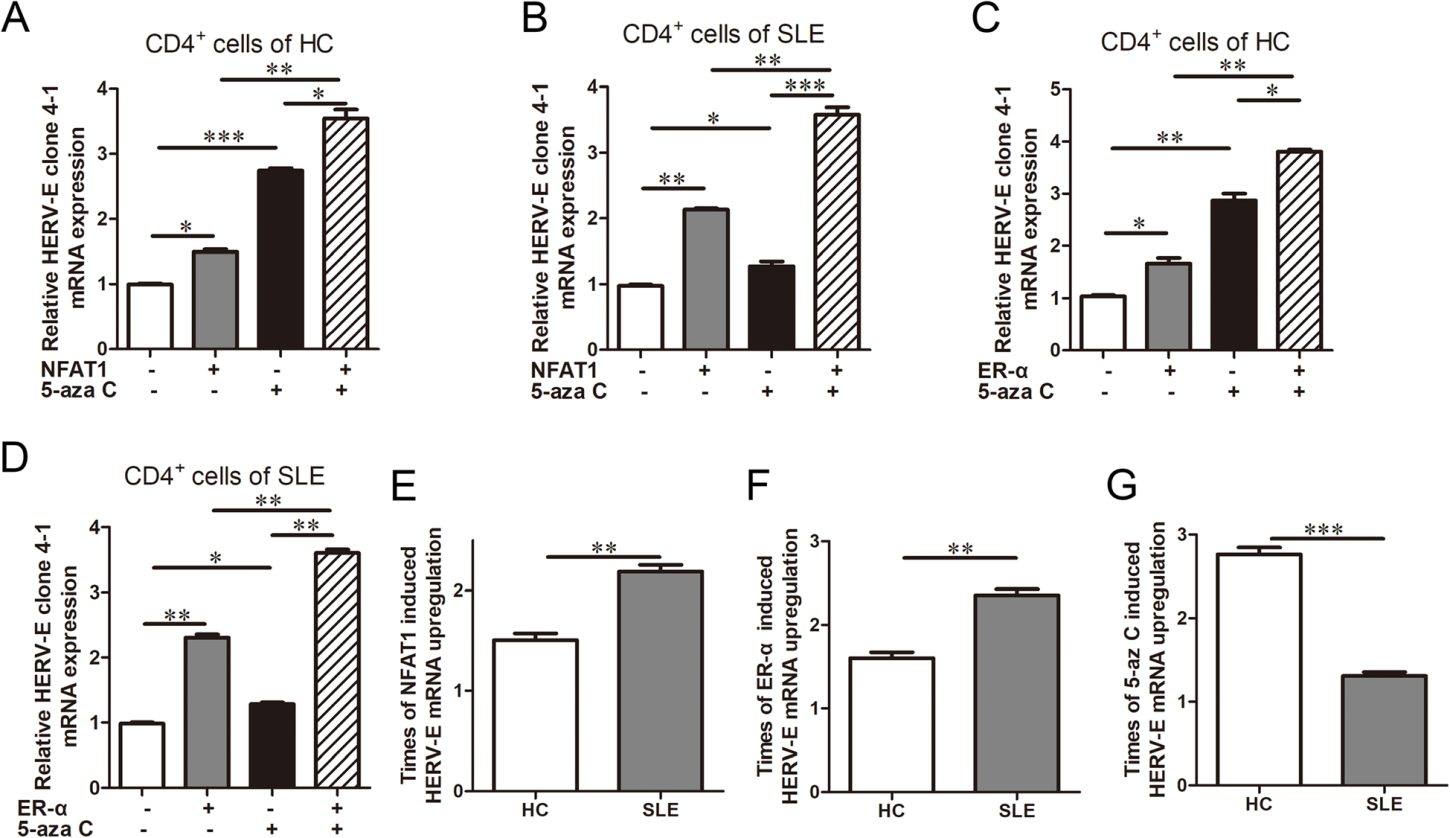
DNA hypomethylation of *HERV-E clone 4–1* 5’LTR contributed to upregulation of *HERV-E clone 4–1* mRNA induced by *NFAT1* and *ER-α***.** CD4^+^ T cells from SLE patient or HC were treated with *NFAT1* plasmids, *ER-α* plasmids, 5-aza C alone or in combination with 5-aza C in vitro. Relative *HERV-E clone 4–1* mRNA expression (**a**-**d**) were detected. **e** and **f** Times of *HERV-E clone 4–1* mRNA upregulation were compared by the Student’s t test in CD4^+^ T cells from SLE patient and HC when *NFAT1* or *ER-α* was overexpressed. **g** Times of *HERV-E clone 4–1* mRNA upregulation were compared by the Student’s t test in CD4^+^ T cells from SLE patient and HC when 5-aza C was used. Data were represented as mean ± SD, *N* = 3. *P < 0.05, **P < 0.01, ***P < 0.001

In our former study, we found the *HERV-E clone 4–1* 5’LTR was hypomethylated in CD4^+^ T cells from SLE patients and its methylation could be inhibited by 5-aza C [7]. Here, we investigated whether this DNA hypomethylation was involved in the *NFAT1* or *ER-α* induced *HERV-E clone 4–1* mRNA upregulation. We found that *HERV-E clone 4–1* mRNA expressions were upregulated when *NFAT1* or *ER-α* was overexpressed or 5-aza C was used in CD4^+^ T cells from SLE patients and HCs (Fig. 4a-d). In CD4^+^ T cells from SLE patients and HCs, *HERV-E clone 4–1* mRNA expressions were higher when both *NFAT1* was overexpressed and 5-aza C was used than that when *NFAT1* was overexpressed or 5-aza C was used (Fig. 4a and b); accordingly, *HERV-E clone 4–1* mRNA expressions were higher when both *ER-α* was overexpressed and 5-aza C was used than that when *ER-α* was overexpressed or 5-aza C was used (Fig. 4c and d). Besides, the times of *HERV-E clone 4–1* mRNA upregulation were higher in CD4^+^ T cells from SLE patients than that of HCs when *NFAT1* or *ER-α* was overexpressed (Fig. 4e and f), and the times of *HERV-E clone 4–1* mRNA upregulation were higher in CD4^+^ T cells from HCs than that of SLE patients when 5-aza C was used (Fig. 4g). These results together suggested that DNA hypomethylation contributed to the upregulation of *HERV-E clone 4–1* mRNA induced by *NFAT1* and *ER-α*.

### *HERV-E clone 4–1* 3’LTR induced DNA hypomethylation and IL-17 release via *miR-302d*/*MBD2*

**Fig. 5:**
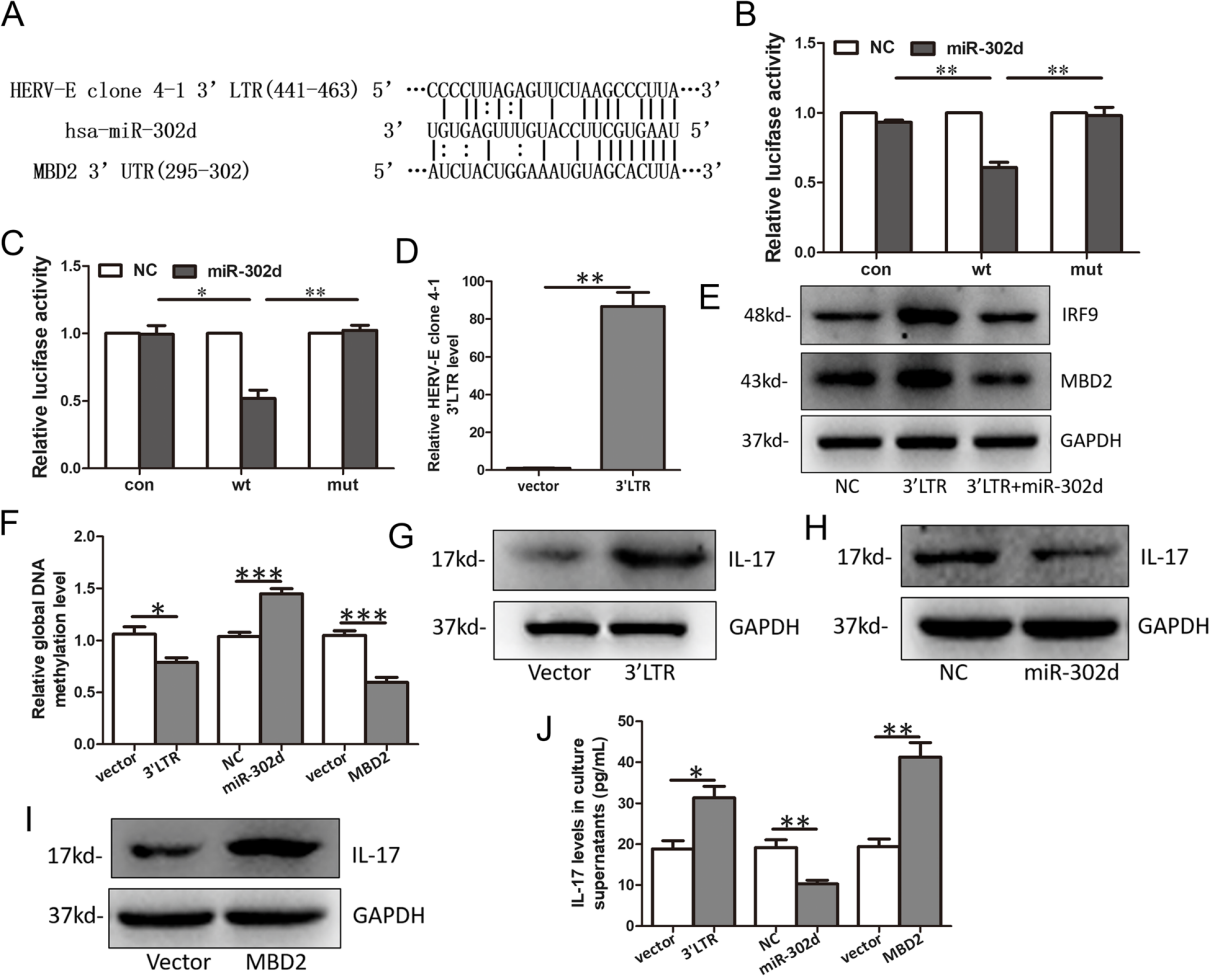
*HERV-E clone 4–1* 3’LTR induced DNA hypomethylation and IL-17 release via *miR-302d*/*MBD2* in CD4+ T cells of SLE. **a** Predicted binding sites of *miR-302d* in the 3’LTR of *HERV-E clone 4–1* mRNA and *MBD2* 3’UTR. **b** and **c** Effects of *miR-302d* on the expression of luciferase reporter genes containing *HERV-E clone 4–1* 3’LTR or *MBD2* 3’UTR. Luciferase activity was normalized to the value obtained in cells transfected with NC oligonucleotides. **d** Relative expression of *HERV-E clone 4–1* 3’LTR mRNA when CD4^+^ T cells were transfected with *HERV-E clone 4–1* 3’LTR expression plasmids. **e** Western blot analysis of IRF9 and *MBD2* proteins in CD4^+^ T cells from SLE patients transfected with *HERV-E clone 4–1* 3’LTR expression plasmids and/or *miR-302d* mimics and the corresponding negative controls. Relative global DNA methylation level (**f**), intracellular IL-17 level (**g**-**i**) and IL-17 level in culture supernatants (**j**) in CD4+ T cells from SLE patient transfected with *HERV-E clone 4–1* 3’LTR expression plasmids, *miR-302d* mimics or *MBD2* expression plasmids and the corresponding negative controls

Since 3’UTRs of mRNAs were reported to act as natural miRNA sponges and could serve as competitive endogenous RNAs (ceRNAs) of other genes through sharing the common miRNAs [17–20]. We want to explore whether the 3’LTR of *HERV-E clone 4–1* mRNA could act as a miRNA sponge and act ceRNAs of other genes. Through programs based on microRNA.org and Targetscan, we found that there was a potential binding site of *miR-302d* in the 3’LTR of *HERV-E clone 4–1* mRNA (Fig. 5a). Then, we performed luciferase reporter assays to determine this interaction. Luciferase assay showed that *miR-302d* mimics could decrease the luciferase activity of reporter containing wt 3’LTR of *HERV-E clone 4–1* while mut binding site attenuated the increase of luciferase activity (Fig. 5b). This suggested that 3’LTR of *HERV-E clone 4–1* could bind to *miR-302d* and act as a sponge for *miR-302d*. We also found *MBD2* was another potential target of *miR-302d* (Fig. 5a) and verified the interaction between *MBD2* 3’UTR and *miR-302d* using luciferase assay (Fig. 5c). Then, we found that overexpression of 3’LTR of *HERV-E clone 4–1* (Fig. 5d) increased the protein levels of *MBD2* and *miR-302d* mimics could rescue the increase of *MBD2* protein by the 3’LTR (Fig. 5e). These results suggested that *HERV-E clone 4–1* acts as a ceRNA of *MBD2* to positively regulate *MBD2* expression in 3’LTR and *miR-302d* dependent manners. We also detected the expression of *IRF-9* which was a proved target of *miR-302d* in SLE [21] and found that overexpression of 3’LTR of *HERV-E clone 4–1* increased the protein levels of *IRF9* and *miR-302d* mimics could rescue the increase of IRF9 protein by the 3’LTR (Fig. 5e).

The mRNA levels of *MBD2* in was increased in CD4^+^ T cells of SLE patients and inversely correlated with global DNA methylation and positively correlated with and SLEDAI score [22, 23]. What’s more, *MBD2* was found to stimulates Th17 cell differentiation and IL-17 release in other autoimmune diseases [24–26] and IL-17 play critical functions in the pathophysiology of SLE [27, 28] So, *MBD2* might play important roles in SLE progression. Then, we intended to further study the role of *HERV-E clone 4–1*, *miR-302d* and *MBD2* in global DNA methylation and IL-17 expression in CD4^+^ T cells of SLE patients. CD4+ T cells were transfected with *HERV-E clone 4–1* 3’LTR expression plasmids, *miR-302d* mimics or *MBD2* expression plasmids. Global DNA methylation levels, intracellular IL-17 level and IL-17 level in culture supernatants were subsequently measured. The results showed that global DNA methylation level decreased when CD4^+^ T cells of SLE were transfected with 3’LTR expression plasmids or *MBD2* expression plasmids and increased when transfected with *miR-302d* mimics (Fig. 5f). Intracellular IL-17 level and IL-17 level in culture supernatants increased when CD4^+^ T cells of SLE were transfected with 3’LTR expression plasmids or *MBD2* expression plasmids and decreased when transfected with *miR-302d* mimics (Fig. 5g-j). All together, these results suggested that *HERV-E clone 4–1* 3’LTR induce DNA hypomethylation and IL-17 release via *miR-302d*/*MBD2* in CD4^+^ T cells of SLE.

## Discussion

Some studies had proved that *HERV-E clone 4–1* mRNA expression was increased in SLE patients, and the expression level of *HERV-E clone 4–1* was associated with SLE disease activity [5–7], however, they didn’t thoroughly investigate the function and mechanism of *HERV-E clone 4–1* in SLE. In this study, we investigated the mechanism of *HERV-E clone 4–1* mRNA upregulation in CD4^+^ T cells from SLE patients and its roles in SLE progression. First, we found *NFAT1* could induce *HERV-E clone 4–1* mRNA expression by binding to its 5′ LTR. *NFAT1*, which is a key factor of Ca^2+^/ calcineurin (CaN)/NFAT signaling pathways, was verified to be activated in SLE [13]. We also demonstrated that *NFAT1* activity was upregulated in SLE and positively correlated with *HERV-E clone 4–1* mRNA expression. *NFAT1* are phosphorylated and reside in the cytoplasm in resting cells; upon stimulation, they are dephosphorylated by calcineurin, translocate to the nucleus, and become transcriptionally active [29–31]. Then the activated *NFAT1* can regulate transcription of some inflammatory cytokines such as IL-6, IL-8, TNF-α and interferon-γ (IFN-γ) [32–35]. Furthermore, we found TNF-α, IL-6, E2, LPS, UVB could upregulate *NFAT1* activity and *HERV-E clone 4–1* mRNA expression and these factors play critical roles in SLE [14, 36–38]. These results together may explain the roles of *NFAT1* in *HERV-E clone 4–1* mRNA expression in SLE.

Adreno cortico hormones are an important class of anti-inflammatory/immunosuppressive drugs. They can inhibit the expression of TNF-α and IL-6 and decrease the activity of SLE [39]. Ca^2+^/CaN/NFAT signaling is an important pathway in the T-cell activation of SLE and some calcineurin inhibitors such as cyclosporine A and tacrolimus have been used in the clinical treatment of SLE [40]. Hydroxychloroquine, which could block Ca^2+^/CaN/NFAT signaling pathway through inhibiting the sustained Ca^2+^ storage release from the endoplasmic reticulum [41], was found to repress *NFAT1* activity and *HERV-E clone 4–1* expression. Prednisolone and hydroxychloroquine are first-line drugs in the treatment of SLE and all the patients followed-up got oral prednisolone and hydroxychloroquine treatment. These reasons interpret it well why *HERV-E clone 4–1* mRNA expressions decreased after prednisolone and hydroxychloroquine treatment. So, we hold that the upregulation of *HERV-E clone 4–1* mRNA is mainly due to the abnormal inflammation / immune status of SLE which involving many inflammatory cytokines and other risk factors. We also found that E2 could upregulate *HERV-E clone 4–1* mRNA expression via *ER-α*. *ER-α* is one of the estrogen receptors which can be activated by estrogen and regulate gene transcription in nucleus [42]. Interestingly, HERV-E was upregulated in breast cancer and ovarian cancer [15, 16] and this probably also has close relationship with E2 and *ER-α*. *ER-α* antagonist is also a good approach to restrain the expression of *HERV-E clone 4–1*. Taken together, we think these signaling pathways are good therapeutic targets for *HERV-E clone 4–1*.

Some studies found the *HERV-E clone 4–1* 5’LTR was hypomethylated in CD4^+^ T cells from SLE patients [7–9]. We found that DNA hypomethylation contributed to upregulation of *HERV-E clone 4–1* mRNA induced by *NFAT1* and *ER-α*. We think DNA hypomethylation of *HERV-E clone 4–1* 5’LTR is an indispensable factor that account for the upregulation of *HERV-E clone 4–1* mRNA for that upregulation of *HERV-E clone 4–1* mRNA mainly exists in SLE while not in some other diseases that involving *NFAT1* and *ER-α* activation.

**Fig. 6:**
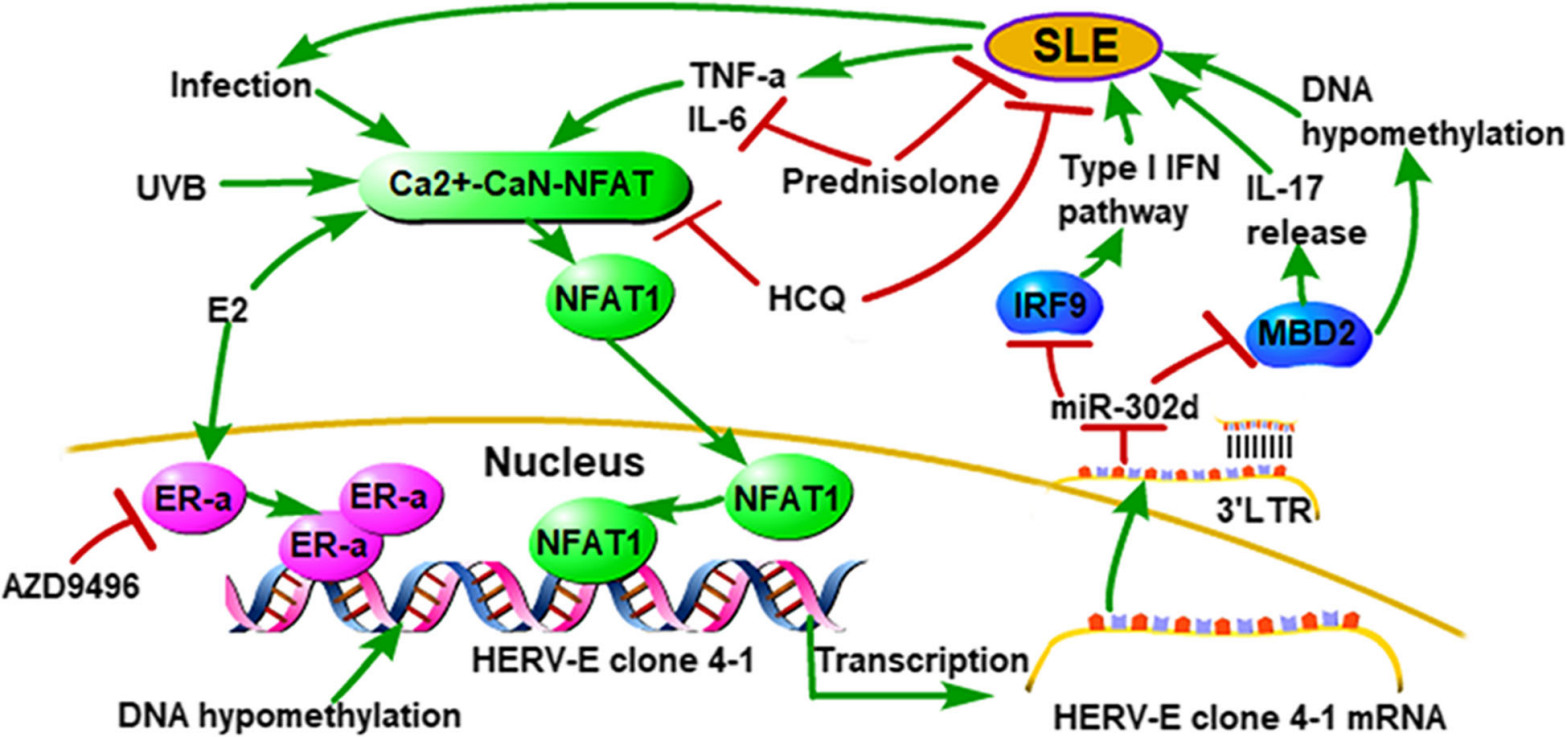
Network diagram hypothesis showing relationship between *HERV-E clone 4–1* and SLE

In this study, we found that *HERV-E clone 4–1* 3’LTR could act as natural miRNA sponges for *miR-302d* to restrain *miR-302d* activity. *MiR-302d* was proved to be downregulated in SLE patient monocytes and could inhibit the type I IFN pathway which was a major contributor to SLE pathogenesis via its target *IRF-9* [21]. *HERV-E clone 4–1* 3’LTR could positively regulate *MBD2* expression by acting as a ceRNA of *MBD2* via *miR-302d* and *HERV-E clone 4–1* 3’LTR could induce DNA hypomethylation and IL-17 release via *miR-302d*/*MBD2* in CD4^+^T cells of SLE. DNA hypomethylation of immune cells in SLE is associated with immune dysfunction and play important roles in the initiation and development of SLE [43, 44]. IL-17 is a proinflammatory cytokine produced by activated T cells and plays a crucial role in disease pathogenesis and represent an attractive therapeutic target for SLE [27, 28]. Thus, we hold that *HERV-E clone 4–1* takes part in disease pathogenesis of SLE through *miR-302d*/*MBD2*/DNA hypomethylation and IL-17 signaling via its 3’LTR. So, *HERV-E clone 4–1* 3’LTR may be a potential therapeutic target of SLE. Taken together, we draw a network diagram hypothesis showing relationship between *HERV-E clone 4–1* and SLE which shows the important roles of *HERV-E clone 4–1* in SLE pathogenesis (Fig. 6).

However, we should admit that we didn’t further investigate the role of *HERV-E clone 4–1* proteins and this is a shortcoming of this study. This mainly because there is no specific antibody for these proteins.

## Conclusions

In conclusion, we found that *HERV-E clone 4–1* mRNA expression was upregulated in CD4^+^ T cells from SLE patients and could act as a good biomarker for diagnosis of SLE. This is associated with the activation of Ca^2+^/CaN/*NFAT1* and E2/*ER-α* signaling pathway and DNA hypomethylation of *HERV-E clone 4–1* 5’LTR. *HERV-E clone 4–1* also takes part in disease pathogenesis of SLE through *miR-302d*/*MBD2*/DNA hypomethylation and IL-17 signaling via its 3’LTR. These signaling pathways may be potential therapeutic targets of SLE.

## Additional file

Additional file 1: **Figure S1**. The structure of HERV-E clone 4–1. **Table S1**. Clinical characteristics of SLE patients and healthy controls. (DOCX 26 kb)

## Data Availability

Not applicable.

## Abbreviations

5-aza C: 5-Azacytidine
AUC: Area Under Curve
CaN: Calcineurin
ChIP: Chromatin immunoprecipitation
E2: 17β-estradiol/estradiol
ER-α: Estrogen receptor-α
HCQ: Hydroxychloroquine
HERV: Human endogenous retroviruses
IFN-γ: Interferon-γ
LPS: Lipopolysaccharides
LTRs: Long terminal repeats
MBD2: Methyl-CpG binding domain protein 2
NFAT: Nuclear factor of activated T cells
ORFs: Open Reading Frames
PBMC: Peripheral blood mononuclear cells
qRT-PCR: Quantitative reverse transcription-PCR
ROC: Operating Characteristic
SLE: Systemic lupus erythematosus
SLEDAI: SLE disease activity index
TNF-α: Tumor necrosis factor-a
UVB: Ultraviolet B
