# Supplementary material for: Increased HERV-E clone 4–1 expression contributes to DNA hypomethylation and IL-17 release from CD4+ T cells via miR-302d/MBD2 in systemic lupus erythematosus
Source: Cell Commun Signal. 2019 Aug 14;17:94. doi: 10.1186/s12964-019-0416-5 (PMC6694475; doi:10.1186/s12964-019-0416-5)
Supplement: Supplementary file 1 — Figure S1. The structure of HERV-E clone 4–1. Table S1. Clinical characteristics of SLE patients and healthy controls. (DOCX 26 kb) [file 12964_2019_416_MOESM1_ESM.docx]

**Figure S1. The structure of HERV-E clone 4-1.**

**
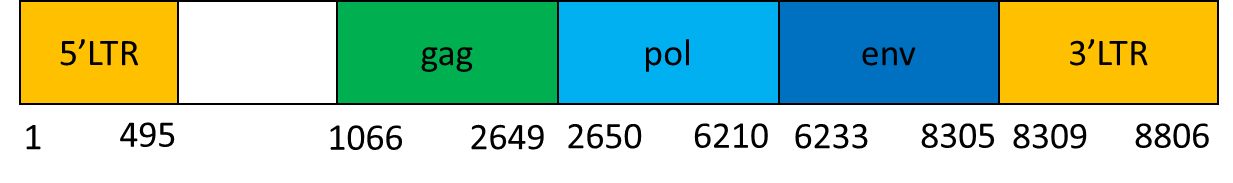
**

**Table S1.** Clinical characteristics of SLE patients and healthy controls.

| Factors | SLE(n=27) | HC(n=21) |
| --- | --- | --- |
| Gender(male/female) | 4/23 | 3/18 |
| Age (years)* | 31.62±6.55 | 31.35±5.93 |
| SLEDAI score* | 8.26±3.27 | NA |

*Data are shown by mean ± SD.
